# A novel assay that characterizes properdin function shows neutrophil-derived properdin has a distinct oligomeric distribution

**DOI:** 10.3389/fimmu.2022.918856

**Published:** 2023-01-12

**Authors:** Sara R. Moore, Smrithi S. Menon, Neeti S. Galwankar, Sadik A. Khuder, Michael K. Pangburn, Viviana P. Ferreira

**Affiliations:** ^1^ Department of Medical Microbiology and Immunology, University of Toledo College of Medicine and Life Sciences, Toledo, OH, United States; ^2^ Department of Medicine, University of Toledo College of Medicine and Life Sciences, Toledo, OH, United States; ^3^ Center for Biomedical Research, University of Texas Health Science Center, Tyler, TX, United States

**Keywords:** complement system, alternative pathway, properdin, neutrophil, oligomers

## Abstract

Properdin acts as an essential positive regulator of the alternative pathway of complement by stabilizing enzymatic convertases. Identical properdin monomers form head-to-tail associations of oligomers in a reported 20:54:26 ratio (most often described as an approximate 1:2:1 ratio) of tetramers (P_4_), trimers (P_3_), and dimers (P_2_), in blood, under normal physiological conditions. Oligomeric size is proportional to properdin function with tetramers being more active, followed by trimers and dimers. Neutrophils are the most abundant granulocyte, are recruited to inflammatory microenvironments, and are a significant source of properdin, yet the ratio of properdin oligomers released from neutrophils is unknown. The oligomer ratio of neutrophil-derived properdin could have functional consequences in local microenvironments where neutrophils are abundant and complement drives inflammation. We investigated the oligomer properties of neutrophil-derived properdin, as compared to that of normal human sera, using a novel ELISA-based method that detects function of properdin in a way that was proportional to the oligomeric size of properdin (i.e., the larger the oligomer, the higher the detected function). Unexpectedly, neutrophil-derived properdin had 5-fold lower function than donor-matched serum-derived properdin. The lower function was due to a lower percentage of tetramers/trimers and more dimers, indicating a significantly different P_4_:P_3_:P_2_ ratio in neutrophil-derived properdin (18:34:48) as compared to donor-matched serum (29:43:29). Release of lower-order oligomers by neutrophils may constitute a novel regulatory mechanism to control the rate of complement activation in cellular microenvironments. Further studies to determine the factors that affect properdin oligomerization and whether, or how, the predominant dimers in neutrophil-derived properdin, assimilate to the ~1:2:1 ratio found in serum are warranted.

## 1 Introduction

The complement system is an essential component of the immune system consisting of three pathways: classical, lectin, and alternative, which are each activated differently, and converge at the generation of C3b. The alternative pathway (AP) is a surveillance system that is always active, whereby C3 in blood spontaneously hydrolyzes to produce C3(H_2_O) (C3 tick-over mechanism) (1). C3(H_2_O) binds Factor B, which is then cleaved by mature Factor D to form a fluid-phase C3 convertase known as C3(H_2_O)Bb (1) that cleaves circulating C3 to C3b. C3b can bind covalently to hydroxyl or amino groups on nearby surfaces where it interacts with Factors B and D to form a surface-bound C3 convertase, C3bBb. C3 convertases cleave C3 to generate C3 fragments (C3a and C3b). It is also argued that the AP is primarily an amplification loop of C3b generated from the classical and lectin pathways with contributions from non-complement proteases (reviewed in (2, 3)). In order for the AP to carry out essential amplification of all three complement pathways, the C3 convertase of the AP is stabilized by a positive regulatory protein, properdin, increasing the half-life of the convertase by 5-10-fold (4, 5). Subsequent binding of C3b near C3 convertases (6) forms C5 convertases that cleave C5, leading to formation of C5a and the membrane-attack complex (C5b-9) (reviewed in (7)). The complement fragments that are generated during complement activation serve as potent pro-inflammatory mediators that are critical for opsonization, immune modulation, chemotaxis, and numerous other functions.

A fine balance between complement activation and regulation ensures the host is protected from danger such as infection, but not overwhelmed by excessive, harmful inflammation. Regulation of the AP by negative regulators, both fluid-phase (AP: Factor H, Factor H-like protein 1; terminal pathway: clusterin, vitronectin) and membrane-bound (AP: CRIg, CD35, CD46, CD55; terminal pathway: CD35, CD46, CD55, CD59), are essential for ensuring complement activity does not result in unintentional damage to the host. In contrast, in order to increase the effectiveness of the AP, properdin serves as an essential positive regulator by 1) stabilizing AP convertases, as mentioned above; 2) enhancing proconvertase (C3bB) formation (8–11); 3) competing with Factor I to prevent cleavage of C3b into the inactive form, iC3b (8, 12–14); and 4) serving as a pattern recognition molecule to initiate AP convertase formation *de novo* by recruiting C3b or C3(H_2_O), followed by Factors B and D (9); however, the relevance of this initiating phenomenon *in vivo* remains unknown. Properdin is an oligomeric glycoprotein, which forms dimers (P_2_), trimers (P_3_), and tetramers (P_4_) of head-to-tail, non-covalent associations of identical monomeric subunits. In healthy serum, these oligomers are present in a stable 20:54:26 ratio of P_4_, P_3_, and P_2_ (15, 16). Each properdin monomer contains 442 amino-acid residues arranged into six complete thrombospondin type 1 repeat (TSR1-6) domains and a N-terminal TGF-β binding (TB) domain (17–19). Properdin oligomers form ring-shaped vertices upon interaction of the TB domain and TSR1 from one monomer with TSR4, TSR5, and TSR6 from another monomer (8, 12, 14). These vertices interact with C3b and Bb of convertases (or Factor B of proconvertases) at a binding site formed by TSR5 and a large loop of TSR6 (8, 11, 12). Properdin oligomerization directly correlates with properdin convertase-stabilizing function (i.e., P_4_ are more functional than P_3_, and P_3_ are more functional than P_2_) (15, 20). Electron microscopy suggests each properdin vertex concurrently binds a convertase, thus delineating the association between oligomer function and its stoichiometry (14). Simultaneous interactions of oligomeric properdin with multiple C3b molecules on an activator cell surface where C3b convertases are forming remains to be determined.

Properdin is present in serum at a concentration of 4-25 μg/ml (15). Unlike most complement proteins which are generated in the liver, properdin is released primarily from activated leukocytes. Cells known to either release or synthesize properdin mRNA include granulocytes, macrophages, monocytes, dendritic cells, primary T cells, mast cells, adipocytes, and endothelial cells (reviewed in (21)). Neutrophils, the most abundant leukocyte, release properdin stored in secondary granules synthesized during bone marrow maturation (22) in response to a variety of inflammatory agonists. Although it is accepted that properdin is present in serum in an approximate, 1:2:1 ratio of P_4_:P_3_:P_2_, the molecular composition of properdin when released by leukocytes is unknown. At local sites of inflammation, where neutrophils are abundant and subject to cytokine stimulation, the distribution of oligomers of neutrophil-derived properdin may further modulate local complement activity, beyond the increased properdin concentration.

In this study, the ability of properdin to capture and generate convertases *de novo* was utilized to develop a novel ELISA-based technique to evaluate the function of properdin in biological samples with a readout that correlated with the oligomeric distribution of properdin in the sample. For the first time, the oligomeric distribution of properdin released from neutrophils was characterized using this assay and confirmed by size exclusion chromatography. The novel assay revealed that the function of neutrophil-derived properdin was significantly lower than donor-matched serum properdin and the decreased function was confirmed to be due to the presence of predominantly P_2_ and P_3_.

## 2 Material and methods

### 2.1 Buffers

The following buffers were used: Gelatin veronal buffer (GVB=; 5 mM veronal, 145 mM NaCl, 0.004% NaN_3_, 0.1% gelatin, pH 7.3), MgEGTA [0.1 M MgCl_2_ and 0.1 M EGTA (ethylene glycol tetraacetic acid), pH 7.4], 0.5 M EDTA (0.25 M EDTA [Ethylenediaminetetraacetic acid] disodium salt-2H_2_O and 0.25 M EDTA tetrasodium salt-2H_2_O, pH 7.4), GVBE (GVB=, 10 mM EDTA), phosphate buffered saline (PBS; 10 mM NaH_2_PO_4_, 145 mM NaCl, pH 7.4), Hanks’ balanced salt solution with Ca^2+^ and Mg^2+^ (HBSS^++^; Gibco), and ELISA dilution buffer (PBS + 0.05% tween + 1% bovine serum albumin (BSA)).

### 2.2 Serum and complement proteins

Normal human serum (NHS) was purchased from Innovative Research or produced by our laboratory using healthy donors. The Institutional Review Board from the University of Toledo College of Medicine and Life Sciences approved the protocols, and written informed consent was obtained from all donors, in accordance with the Declaration of Helsinki. Properdin-depleted and C3-depleted serum were purchased from Complement Technology Inc. Unfractionated pure properdin was purified from normal human plasma as described previously (23). Rabbit erythrocytes (E_R_) were prepared from blood obtained from Rockland Immunochemicals.

### 2.3 Antibodies

The following antibodies were used: mouse monoclonal immunoglobulin G (IgG)1 anti-properdin 3A3E1 (biotin-labeled) and 6E9E6 (unlabeled) antibodies developed by our laboratory as previously described (24) and biotinylated mouse IgG1 anti-human C3/C3b/iC3b monoclonal (clone 7C12) (Cedarlane).

### 2.4 Human neutrophil cell isolation and activation

Human whole blood was collected *via* venipuncture from healthy donors. The Institutional Review Board from the University of Toledo College of Medicine and Life Sciences approved the protocols, and written informed consent was obtained from all donors, in accordance with the Declaration of Helsinki. Blood was drawn into BD vacutainer tubes containing 12 mg K3 (tripotassium) EDTA and polymorphonuclear (PMN) cells were isolated by using a Polymorphprep™ gradient solution (Axis Shield) following manufacturer’s instructions. PMN cells (5.0 x 10^7^ cells/ml) in 0.2% BSA/HBSS++ buffer, were activated using 20 ng/ml of phorbol 12-myristate 13-acetate (PMA) (Enzo Life Science) for 30 min at 37°C. The PMNs were centrifuged at 600 x g for 10 min. The supernatant was removed and centrifuged at 13,000 g for 2 min to remove cell debris. PMNs without PMA treatment were prepared as a control. The supernatants from PMA-activated and non-activated PMNs were assayed for properdin concentration, as indicated below.

### 2.5 Sandwich ELISA to quantify properdin concentration

Properdin concentration of test samples was assayed by a sandwich ELISA as previously reported by our laboratory (25). The samples (i.e., fractions collected from: pure properdin, NHS, neutrophil supernatant, and donor-matched serum) were tested at varying dilutions in ELISA dilution buffer, as indicated in the figure legends. Other samples tested, but not shown, include neutrophil supernatant samples (diluted 1/80, 1/160, and 1/320), donor-matched serum, NHS, and C3-depleted serum (diluted 1/800, 1/1600, 1/3200, and 1/6400).

### 2.6 Separation of properdin oligomers from native properdin and biological samples

Physiological oligomeric forms of properdin P_2_, P_3_, P_4_, and non-physiological aggregated properdin (P_n_) were isolated from purified properdin by size exclusion chromatography as previously reported with minor modifications (23). Briefly, 200 μg purified properdin in a total volume of 400 μl in PBS was loaded onto a Phenomenex BioSep 5 μM SEC s4000 500Å liquid chromatography column (600 x 7.8 mm) with guard column (75 x 7.8 mm) and was eluted at a flow rate of 0.3 ml/min in PBS. Fractions were collected in 250 μl volumes in glass tubes (12 x 75 mm) that were previously blocked with 500 μl GVB= overnight at 4°C and then emptied prior to sample collection. Collected fractions were stored at 4°C and used within one week of separation. Similarly, physiological oligomeric forms of properdin were separated from NHS and neutrophil supernatant by size exclusion chromatography by diluting the sample to contain 4 μg or 150 ng properdin (properdin concentration determined by sandwich ELISA) with 1% BSA and 20 mM EDTA in PBS, and syringe-filtered using 0.20 μM regenerated cellulose filter membranes (Phenomenex). Samples (400 μl final volume) were loaded onto a size chromatography column as described for purified properdin. For all samples that were run through the size exclusion column, the concentration of properdin within each fraction was determined using the sandwich ELISA described in section 2.5. A visual representation of the ratio of properdin oligomers in the sample was constructed by graphing the properdin concentration of each fraction. Peak fractions and no more than 3 fractions to the left or right of the peak fraction were selected for further analyses (i.e., properdin functional ELISA, hemolytic assay) of purified oligomers.

### 2.7 Quantification of properdin oligomeric ratios in biological specimens

**Figure 1:**
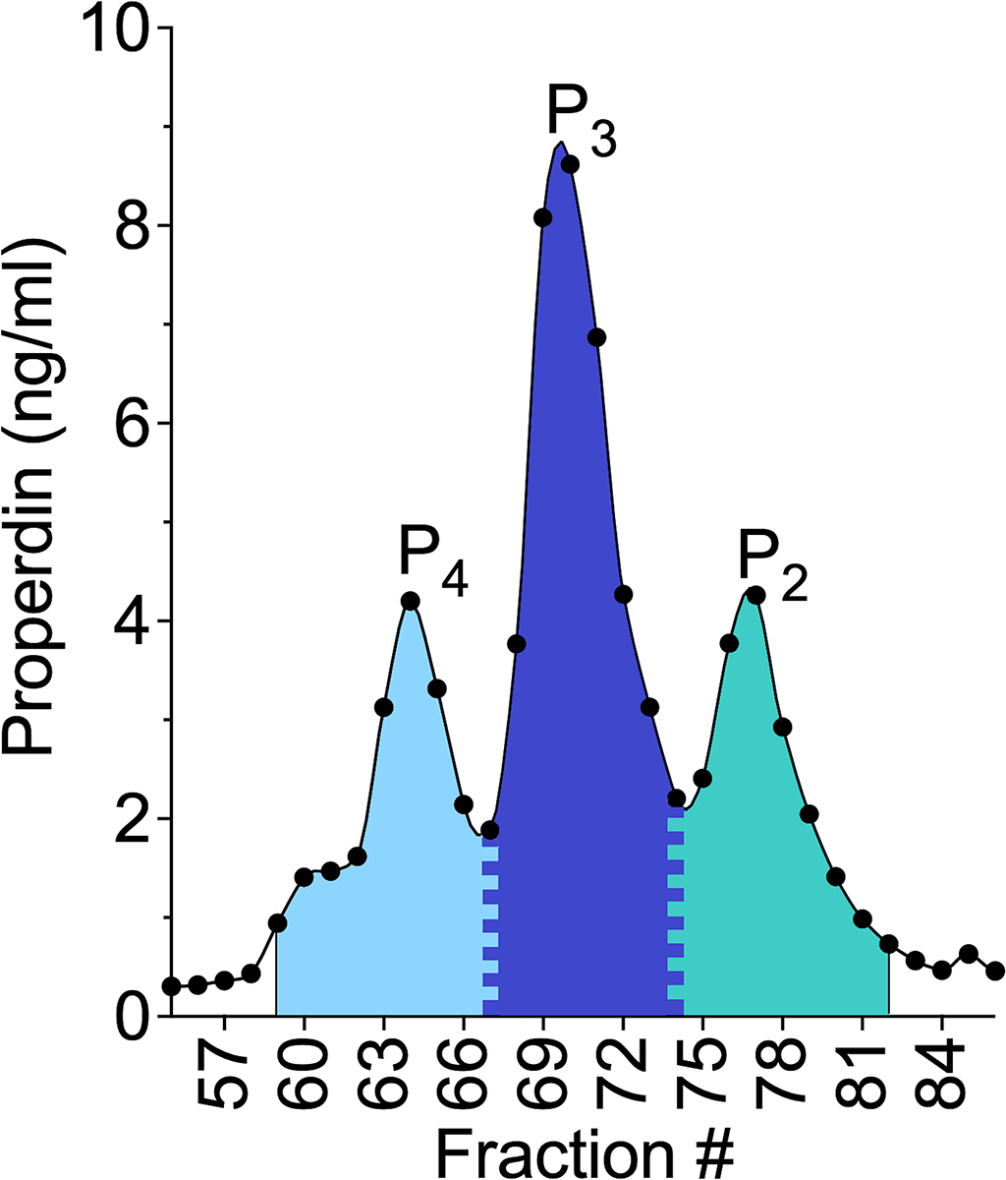
Visual representation of the method for determining the ratio of properdin oligomers using the properdin sandwich ELISA data from the fractions collected after performing size exclusion chromatography on NHS, from donor #4 as described in “Materials and Methods” (section 2.7);. The width of the P_4_ peak was considered five fractions to the left of the P_4_ top fraction and all fractions to the right of the top fraction up until the fraction present in the valley between the P_4_ and P_3_. The fraction present at the valley between peaks was assigned to both P_4_ and P_3_ by dividing the concentration of that fraction equally between the two properdin oligomers. The width of the dimer peak was considered five fractions to the right of the P_2_ top fraction and all fractions to the left up until the valley between the P_2_ and P_3_ peaks. The fraction present at the valley between these peaks was assigned to both P_2_ and P_3_ by dividing the concentration of that fraction equally between the two properdin oligomers. The width of the P_3_ peak was defined as all fractions in between the P_4_/P_3_ valley and the P_3_/P_2_ valley. After assigning fractions to properdin oligomers, the concentration from fractions belonging to each properdin oligomer form was totaled. Then, all fractions, from all oligomers were summed for a grand total. The ratio of properdin oligomers was determined by dividing the total concentration of each properdin oligomer by the grand total and multiplying by 100.

A systematic approach for determining the fraction boundaries of the properdin oligomer forms within each sample was applied using the plotted concentration data (method described in section 2.5) for each fraction. **Figure 1** shows properdin concentration in each fraction derived from size exclusion chromatography of NHS (from donor #4). The width of the P_4_ peak was considered five fractions to the left of the P_4_ top fraction and all fractions to the right of the top fraction up until the fraction present in the valley between the P_4_ and P_3_. The fraction present at the valley between peaks was assigned to both P_4_ and P_3_ by dividing the concentration of that fraction equally between the two properdin oligomers. The width of the P_2_ peak was considered five fractions to the right of the P_2_ top fraction and all fractions to the left up until the valley between the P_2_ and P_3_ peaks. The fraction present at the valley between these peaks was assigned to both P_2_ and P_3_ by dividing the concentration of that fraction equally between the two properdin oligomers. The width of the P_3_ peak was defined as all fractions in between the P_4_/P_3_ valley and the P_3_/P_2_ valley. After assigning fractions to properdin oligomers, the concentration from fractions belonging to each properdin oligomer form was totaled. Then, all fractions, from all oligomers were summed for a grand total. The ratio of properdin oligomers was determined by dividing the total concentration of each properdin oligomer by the grand total and multiplying by 100. Comparable results were obtained when fitting the concentration data described in **Figure 1** to three peaks using computer-generated curves applying Fityk software (26) assuming Gaussian distributions for each species (data not shown).

### 2.8 Development of an ELISA-based assay to assess properdin function

The following assay parameters were varied and overall optimal conditions allowing the greatest signal to noise ratio were chosen (data not shown): concentration of sensitizing anti-properdin monoclonal antibody 6E9E6, properdin concentration in the sample, concentration of properdin-depleted sera, properdin function reaction time, concentration of anti-C3b monoclonal antibody, dilution of horseradish peroxidase-streptavidin, and absorbance read time. Two versions of the assay that result in equivalent outcomes (using either medium-binding full-area or high-binding half-area ELISA plates) were standardized and carried out as described below.

#### 2.8.1 Properdin functional ELISA to assess properdin function in purified properdin and in biological samples

A 96-well, high-binding, half-area, ELISA plate (Greiner Bio One) was coated with 50 μl/well of 1 μg/ml non-inhibitory anti-properdin monoclonal antibody 6E9E6 diluted in PBS overnight at 4°C. The plate was washed 4x with 130 μl/well 1x PBS containing 0.05% Tween-20 (PBST) and blocked for 2 h at 37°C with 125 μl/well 3% BSA in PBS. Properdin sources were diluted to equivalent concentrations within each assay (ng/ml; indicated in figures) in ELISA dilution buffer and 20 mM EDTA then added to the plate and incubated at 37°C for 1 h for properdin capture by the coating antibody, 6E9E6. Background was determined by adding properdin-depleted serum (for serum), PBS (for pure properdin), or 0.2% BSA with HBSS++ (for neutrophil supernatant), instead of a properdin source. The plate was washed 4x with 130 μl/well PBST and while on ice, 50 μl/well properdin-depleted serum diluted 1/10 in GVB= with 5 mM MgEGTA was added to the plate while cold water was added to empty wells not containing any sample to ensure even temperature distribution across the plate. The plate was incubated for 45 min at 37°C. 12.5 μl/well cold 0.1 M EDTA was added to all wells to stop complement activity, and the plate was washed 4x with 130 μl/well PBST. C3b deposited covalently on the plate was detected with 50 μl/well biotinylated anti-C3b monoclonal antibody at 200 ng/ml for 1 h at 37°C. The plate was washed 4x with PBST and incubated for 45 min at 37°C with horseradish peroxidase-streptavidin (Cedarlane) diluted 1:2500 in ELISA dilution buffer. The plate was washed 4x with 130 μl/well PBST and incubated for 30 min with 10-parts ABTS to 1-part ABTS enhancer at room temperature and absorbance was read at 405 nM on a Tecan Infinite M200 spectrophotometer. During each incubation, the plate was sealed with a plastic film. Data was normalized by dividing C3b deposition of each sample by C3b deposition of an internal plate control, NHS.

#### 2.8.2 Properdin functional ELISA to assess properdin function in C3-depleted serum, and neutrophil supernatant and serum matched samples

This assay used full-area, medium-binding plates (Greiner Bio One), resulting in differences in some of the ELISA parameters as compared to section 2.8.1 as follows: (a) all volumes at all steps were doubled; (b) the plates were coated with 10 ug/ml of the 6E9E6 antibody; (c) the following sources of properdin were used: C3-depleted serum, NHS, neutrophil supernatant, or donor-matched serum; (d) properdin-depleted serum was used at 1/10-1/16 dilution and the incubation that followed was 90 min; (e) the biotinylated anti-C3b antibody was used at 20 ng/ml; and (f) the horseradish peroxidase-streptavidin was diluted 1/5000.

### 2.9 Properdin functional hemolytic assay to assess properdin function in purified properdin oligomers and in biological samples

2 x 10^7^ E_R_ were mixed with GVB= and the following reagents at the indicated final concentrations: 2.5 mM MgEGTA or 10 mM EDTA, 0-60 ng/ml of properdin from NHS, pure properdin, or properdin oligomers from pure properdin (P_2_, P_3,_ and P_4_ and P_n_), and 20% properdin depleted serum in a total 50 μl volume. Next, the mix was incubated for 10 min at 37°C, mixing the tubes every 5 min. The tubes were then placed on ice and 200 μl of cold GVBE was added to each tube to stop the reaction. The tubes were spun at 2000 g for 3 min at 4°C. The absorbance of 200 μl of each supernatant was measured in a microtiter plate at 414 nM. The % of hemolysis was calculated using the formula: [(A414-background A414 in the presence of EDTA)/(maximum A414 determined by water lysis-background A414 in the presence of EDTA)] x 100.

### 2.10 Statistical analyses

Data was analyzed by unpaired t-test, one-way ANOVA with Tukey’s post-test, or two-way ANOVA with Bonferroni’s post-test using GraphPad Prism version 9.2.0 for Mac OS X (GraphPad Software, San Diego, California, USA), or by ANCOVA analysis followed by Tukey’s *post hoc* test using SAS 9.4 (SAS Institute Inc., Cary, NC, USA) (as indicated in figure legends or results section). P values less than 0.05 were considered significant.

## 3 Results

### 3.1 Development of an ELISA-based method to characterize properdin function in biological samples

**Figure 2:**
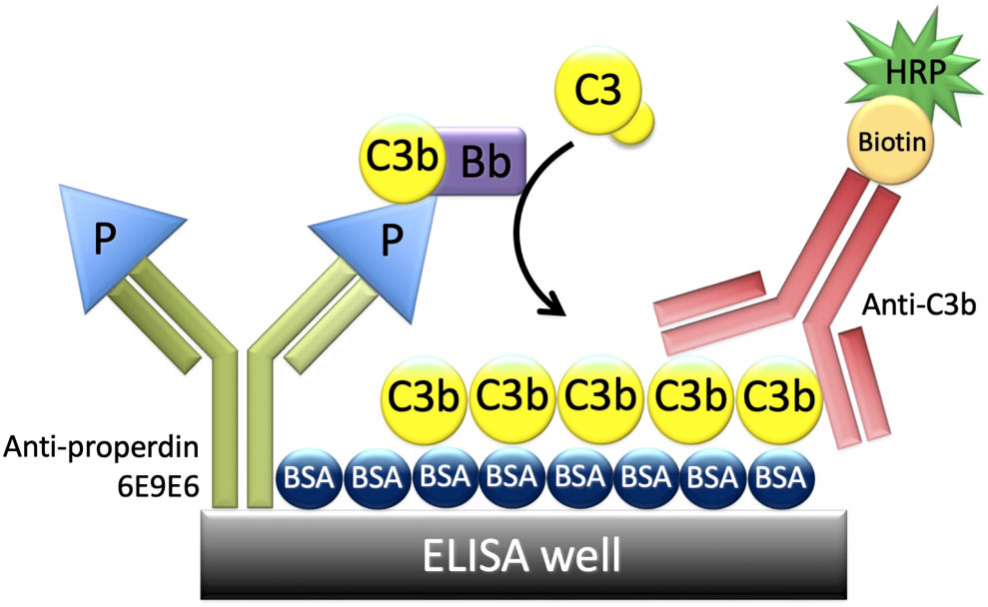
Principle of the properdin functional ELISA. An ELISA plate was coated with a non-inhibitory anti-properdin monoclonal antibody 6E9E6 (in green), blocked with BSA, and then properdin was captured from various sources (purified properdin, serum, neutrophil supernatant). The plate was washed, and properdin-depleted serum was added as a source of complement proteins including C3b, C3(H_2_O), Factor B, and Factor D. C3b or C3(H_2_O) and Factor B bind to properdin to form C3 convertases, C3bBb, or C3(H_2_O)Bb. Convertases cleave C3 and C3b is deposited covalently on BSA and detected with biotin-labeled anti-C3b (in red) followed by horseradish peroxidase-streptavidin, resulting in a colorimetric reaction.

Given that properdin oligomer distribution influences overall properdin function (P_4_ have greater function than P_3_, and P_3_ have greater function than P_2_), an assay that could detect functional differences in biological samples was developed. This was accomplished using a multiplexed (96-well format), ELISA-based approach (**Figure 2**). In order to measure the function of properdin captured from a biological source, a non-inhibitory, anti-properdin monoclonal antibody (6E9E6) was used as a capture antibody. In addition, to ensure that equivalent amounts of properdin (ng/ml) could be captured, irrespective of the oligomerization state, 6E9E6 was used because it did not detect differences between P_2_, P_3_, and, P_4_ (24). Before the next step, the concentration of properdin in the samples was determined using the sandwich ELISA described in the “Materials and Methods” (section 2.5). The next step of the functional properdin ELISA consisted in adding to the 6E9E6-sensitized wells, equivalent concentrations of properdin within biological samples (i.e., serum, neutrophil supernatant) or pure properdin, in the presence of EDTA (to prevent complement activation). After properdin capture, the plate was washed, so that only captured properdin remained, and incubated with properdin-depleted serum with MgEGTA (to facilitate AP activity only). Using properdin-depleted serum as a source of complement proteins for *de novo* convertase formation on the captured properdin, ensured properdin activity was only contributed by the captured properdin. The efficiency of properdin to initiate AP activity (i.e., properdin function) was determined by the level of C3b covalently bound to the BSA coating the plate, which was detected by an anti-C3b monoclonal antibody. The inter- and intra-assay coefficient of variation (CV) for the assay format described in “Materials and Methods” section 2.8.1, was 19.6% and 5.3%, respectively. For the format described in section 2.8.2, the inter-assay and intra-assay CV was 9.7% and 6.5%, respectively. Additional validation is described below.

#### 3.1.1 The properdin functional ELISA can detect differences between properdin oligomers

**Figure 3:**
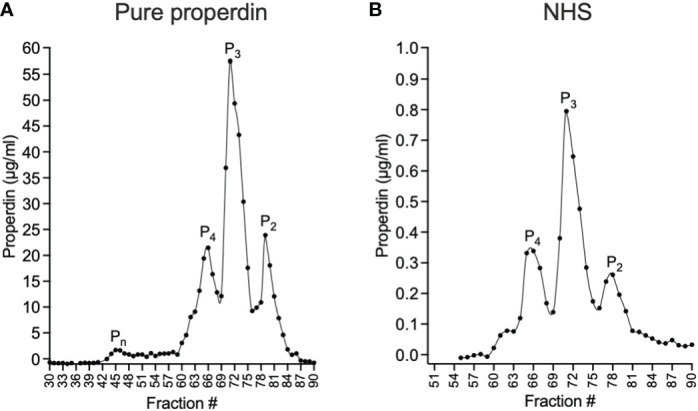
Separation of properdin forms in purified properdin and NHS by size exclusion chromatography and quantification of properdin in the fractions by sandwich ELISA. Properdin from a pure, unfractionated properdin preparation **(A)** or in NHS **(B)** was separated into tetramers (P_4_), trimers (P_3_), dimers (P_2_) and non-physiological aggregates (P_n_) by size exclusion chromatography and the concentration of properdin in the collected fractions (diluted 1:3200 **(A)** and 1:100 **(B)**) was determined by sandwich ELISA as described in “Materials and Methods” (section 2.5 and 2.6) and graphed.

The functional ELISA was validated by evaluating the ability of different captured properdin oligomers to promote C3b deposition. For this purpose, size exclusion chromatography was used to separate physiological oligomeric forms (P_2_, P_3_, and P_4_) and non-physiological aggregates (P_n_) from unfractionated pure properdin. Physiological oligomers in the context of NHS were also separated. After fractionating the samples containing properdin, the concentration of properdin in the collected fractions was determined by sandwich ELISA and the resulting concentrations were plotted to visualize the oligomer distribution in the sample for purified properdin (**Figure 3A**) and NHS (**Figure 3B**). Both properdin sources showed P_4_, P_3_, and P_2_ peaks at similar ratios (i.e., ~18:57:25 for unfractionated pure properdin and ~20:52:27 for NHS) and the unfractionated pure properdin contained a small P_n_ peak.

**Figure 4:**
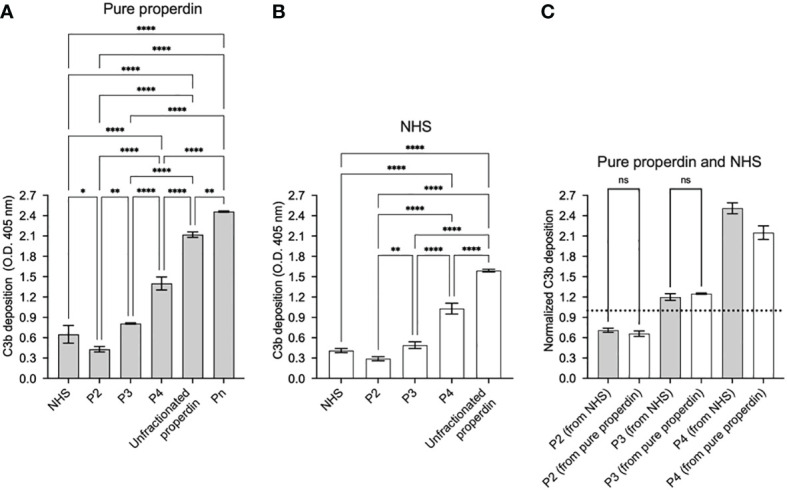
Properdin functional ELISA can distinguish function between properdin oligomers derived from pure properdin and serum. Properdin function was quantified by the properdin functional ELISA where the readout was C3b deposition as described in “Materials and Methods” (section 2.8.1). Data is graphed minus background of samples containing no properdin during the properdin capture step. All samples were tested at equivalent concentrations (200 ng/ml). Properdin function was quantified in properdin oligomer samples (P_n_, P_4_, P_3_, and P_2_) that were separated from pure properdin **(A)** and in P_4_, P_3_, and P_2_ from NHS **(B)**. Both **(A)** and **(B)** include assessment of function of unfractionated NHS and unfractionated pure properdin as controls. The data is a representative of 3 experiments and is graphed as mean and standard deviation of triplicates. **(C)** C3b deposition normalized to an internal plate control (NHS, dotted line) for P_2_, P_3_, and P_4_ derived from pure properdin (as shown in **A**) and NHS (as shown in **B**). Significant differences in C3b deposition between samples was assessed by one-way ANOVA with Tukey’s multiple comparison test; p<0.0001 ****, p<0.01 **, p<0.05 *, p≥ 0.05 non-significant (only shown for panel **C**).

The function in the selected chromatography fractions of each separated properdin oligomers were assayed at equivalent concentrations in the functional ELISA as described in “Materials and Methods” (section 2.8.1). The assay detected significant differences in function between individual properdin oligomers separated from both pure properdin and serum. P_2_ from purified properdin were 30% and 53% as active as P_4_ and P_3_ from purified properdin, respectively. In the case of oligomers separated from serum, P_2_ were 37% and 64% as active as P_4_ and P_3_, respectively. P_3_ from pure properdin and serum were 58% as active as P_4_ from pure properdin and serum. The function of properdin in NHS was closest to pure P_3_ and physiological P_2_/P_3_, as expected given NHS lacks the P_n_ forms (15, 27). P_n_, which are the highly aggregated, non-physiological oligomers of properdin, showed the highest function, and unfractionated pure properdin, which contains P_n_ and the physiological oligomers, resulted in the second highest function (**Figures 4A, B**).

**Figure 5:**
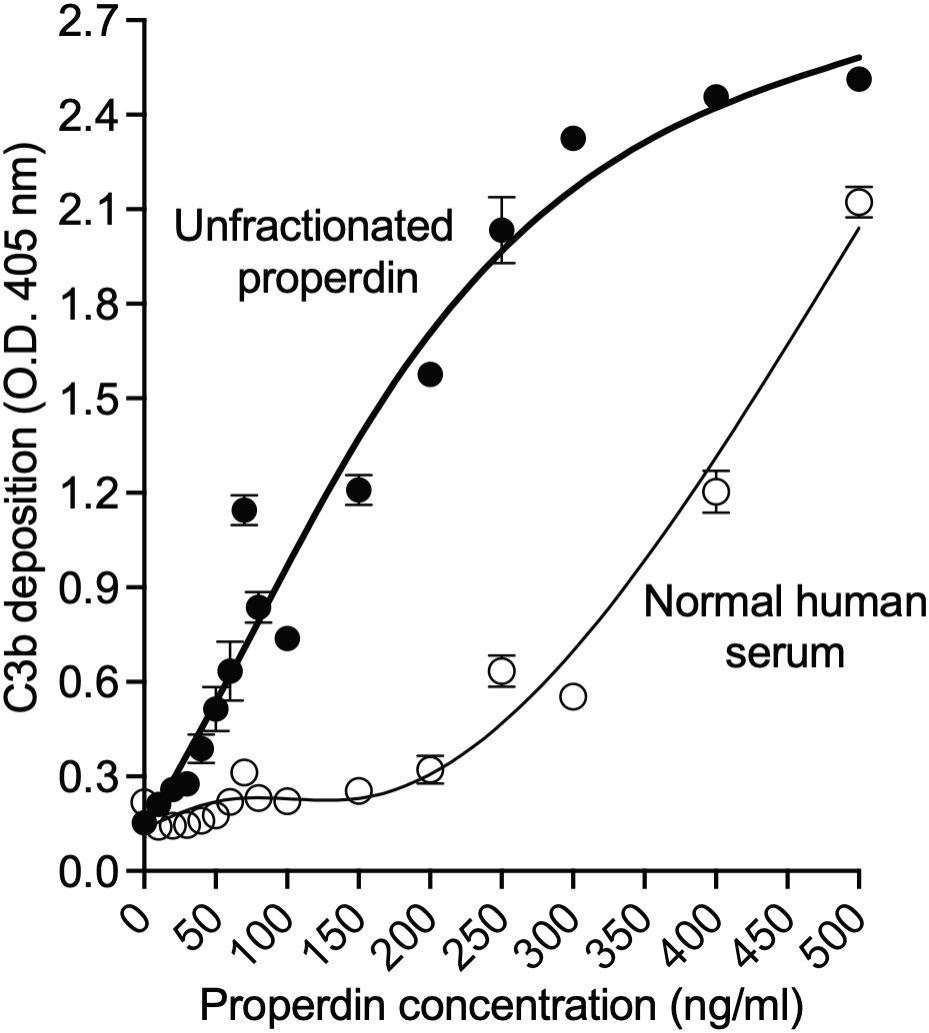
Properdin functional assay detected dose-dependent increases in properdin function and wide functional differences between samples with distinct oligomeric composition. Properdin concentration of unfractionated pure properdin and of properdin in the context of NHS was determined by sandwich ELISA as described in “Materials and Methods” (section 2.5), followed by assessment of function in the samples using the properdin functional ELISA as described in Materials and Methods (section 2.8.1). Unfractionated pure properdin and properdin in NHS were assessed at varying properdin concentrations, as indicated in the x-axis. Data was graphed as mean and standard deviation of triplicates from one experiment.

The utility of the functional ELISA to detect dose-dependent functional differences between unfractionated pure properdin that has P_2_, P_3_, P_4_, and aggregated P_n_ forms, and serum-derived properdin that lacks P_n_ forms was also tested. The function of 16 concentrations (0-500 ng/ml) of unfractionated pure properdin and properdin within serum was assessed. This resulted in a concentration-dependent increase in both serum-derived and unfractionated pure properdin with properdin in serum having lower function at the tested doses (**Figure 5**), indicating that the properdin functional assay detected dose-dependent increases in properdin function and wide functional differences between samples with distinct oligomeric composition.

#### 3.1.2 The hemolytic properdin functional assay does not detect greater properdin function when higher order properdin oligomers are present

**Figure 6:**
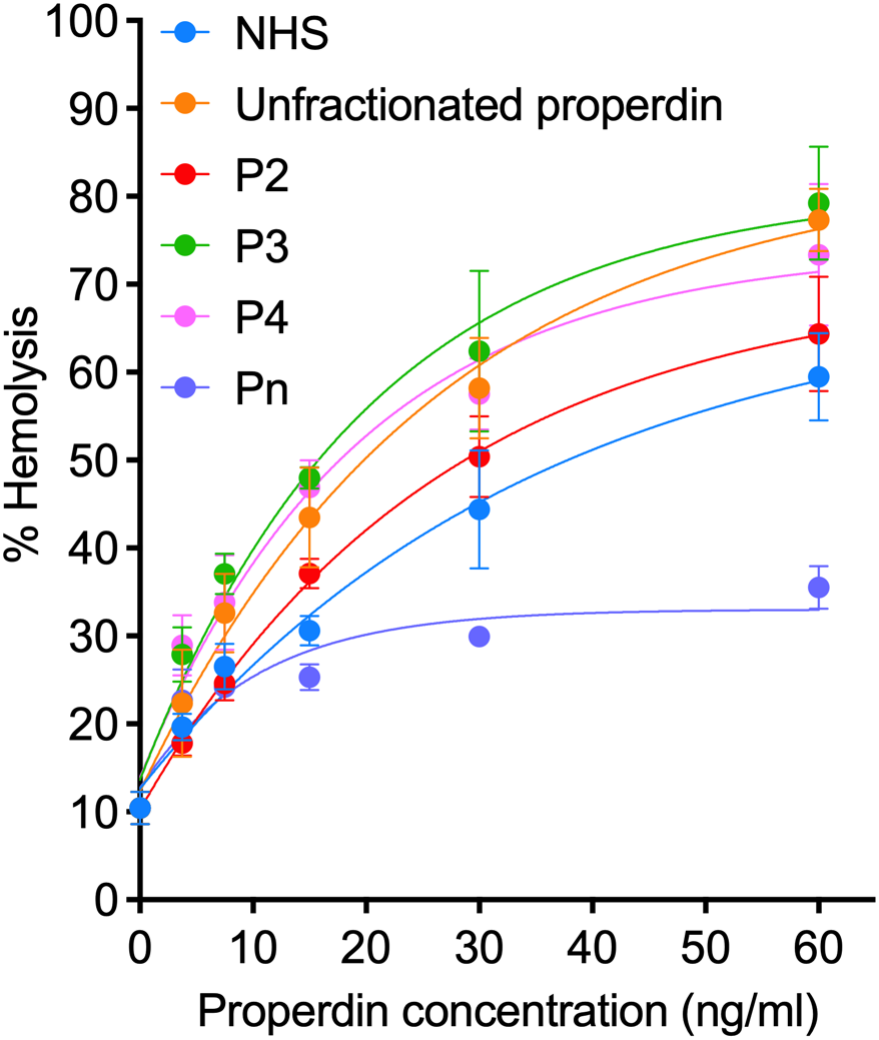
Properdin functional hemolytic assay does not detect greater function in higher order oligomers. Properdin function was quantified in unfractionated pure properdin and NHS, along with properdin oligomers separated from pure properdin (P_n_, P_4_, P_3_, and P_2_), using a properdin functional hemolytic assay where % hemolysis is the read out as described in “Materials and Methods” (section 2.9). Data was graphed as mean and standard deviation of two independent experiments with duplicates.

To determine if another assay, in addition to the functional ELISA, can detect functional differences in samples with distinct oligomer composition, we also evaluated the function of unfractionated pure properdin, properdin in NHS, and properdin in individual properdin oligomers (P_2_, P_3_, P_4_, and P_n_) in a properdin functional hemolytic assay using E_R_, which are susceptible to AP lysis (**Figure 6**). Using ANCOVA analysis with Tukey’s *post hoc* test, the data indicate that although NHS has similar function as P_2_ at all concentrations tested, no differences were observed between unfractionated pure properdin (that contains contaminating P_n_) and P_3_, and P_4_ had the same activity as P_3_. In addition, P_n_ resulted in the lowest hemolysis, most likely due to consumption of complement in the serum, as described previously for high order properdin oligomers (15). Overall, the data indicates that under the conditions tested, the hemolytic assay cannot detect oligomer-dependent differences in properdin function.

#### 3.1.3 Properdin functional ELISA does not capture serum-derived properdin in combination with C3(H_2_O) or C3b

**Figure 7:**
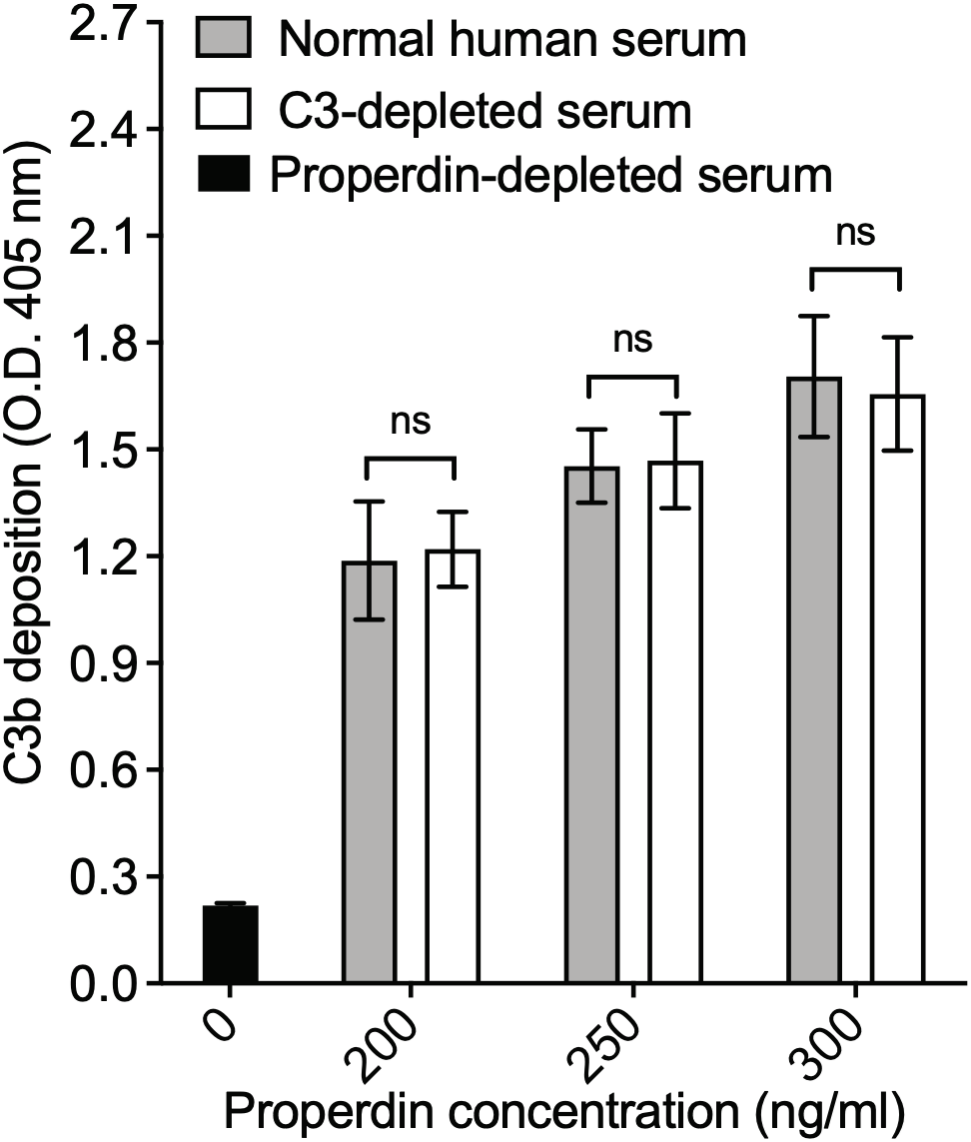
Properdin functional ELISA does not capture properdin in combination with C3 components. Properdin function was quantified by the properdin functional ELISA where the readout is C3b deposition as described in “Materials and Methods” section 2.8.2. Data is graphed minus background of samples containing no properdin during the properdin capture step. All samples were tested at equivalent concentrations (200-300 ng/ml). Results are shown as mean and standard deviation of two independent experiments with triplicates. Significant differences in C3b deposition between samples was assessed by two-way ANOVA with Bonferroni *post hoc* test where p≥ 0.05 non-significant (ns).

The readout of the functional ELISA is the deposition of C3b generated by C3 convertases initiated by properdin. A previous report described that properdin can be found complexed to certain proteins in serum, such as C3(H_2_O), C3 fragments, clusterin, IgG, and C3b-C3b-IgG complexes (28). In order to rule out interference from C3b deposition being potentially contributed by C3(H_2_0) or C3b associated with properdin during the step when properdin was captured from serum, we compared the function of properdin within NHS to properdin captured from C3-depleted serum using the functional ELISA as described in the “Materials and Methods” (section 2.8.2). The assay detected no differences in function between serum-derived properdin and properdin derived from C3-depleted serum at the tested concentrations of properdin (**Figure 7**), indicating that if any C3(H_2_O) or C3b was being captured with the properdin it does not interfere in the assay itself.

### 3.2 Assessment of neutrophil-derived properdin oligomeric distribution

#### 3.2.1 Human neutrophil-derived properdin has lower function than properdin in donor-matched serum as detected by the properdin functional ELISA

**Figure 8:**
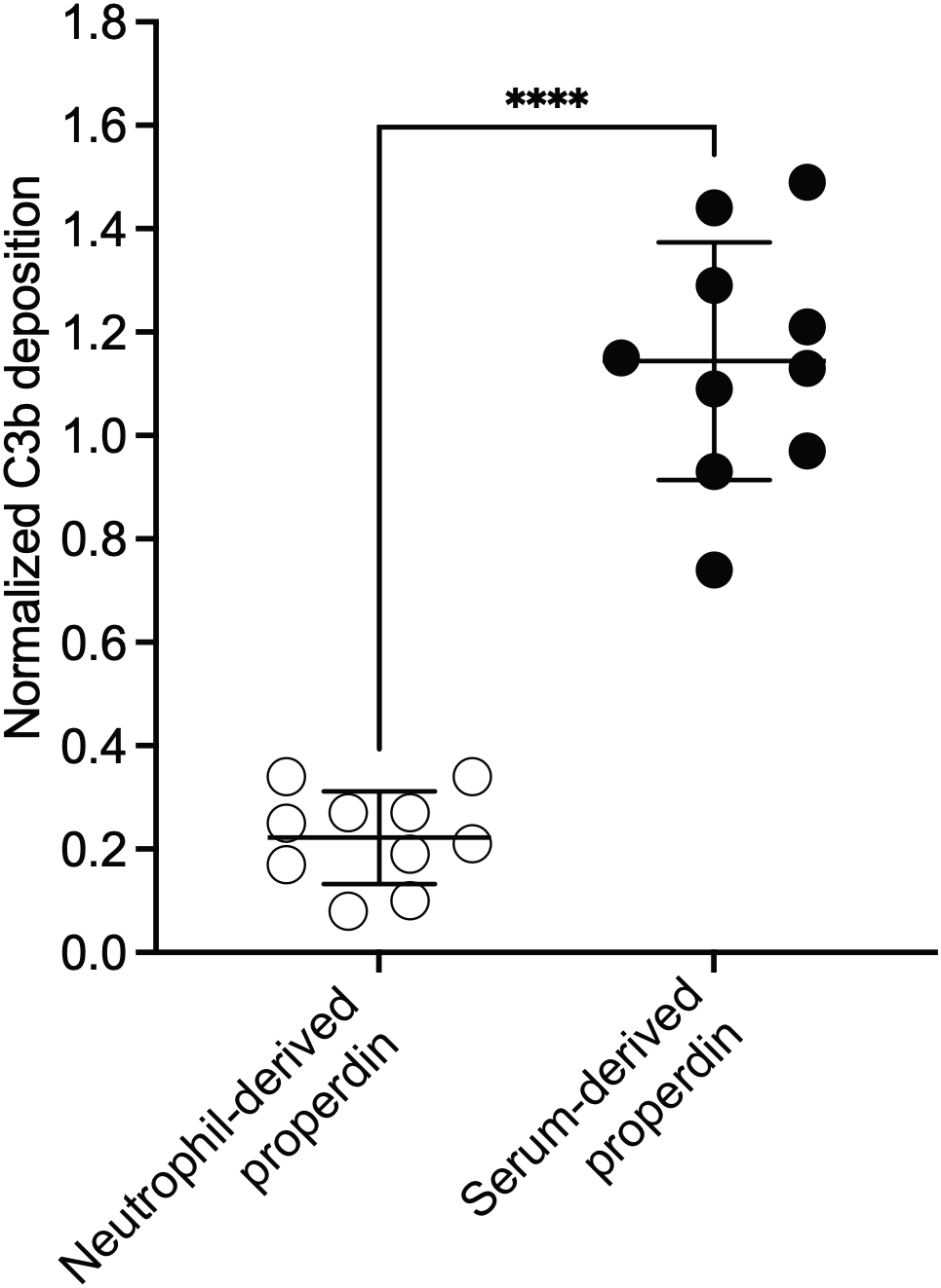
Neutrophil-derived properdin had significantly lower function than serum-derived properdin, when assessed in the properdin functional ELISA, at equivalent concentrations. Properdin function was quantified by properdin functional ELISA where the readout was C3b deposition as described in “Materials and Methods” (section 2.8.2). Data is graphed minus background of samples containing no properdin during the properdin capture step. Properdin function in phorbol-12-myristate-13-acetate (PMA) activated neutrophil supernatant and serum collected from 10 healthy donors was quantified. All samples were tested at equivalent concentrations (300 ng/ml). Results are shown as mean and standard deviation from two independent experiments with triplicates. Significant differences in C3b deposition between samples was assessed by unpaired t test; p< 0.0001****.

The ability of the functional ELISA to detect functional differences between samples that contain different oligomer composition (e.g., between NHS and pure properdin or individual oligomers), suggested that the assay can serve as a first indication of potential differences in distribution of properdin oligomers within a sample. Thus, the assay was used to screen the function of properdin within activated neutrophil supernatants isolated from the blood of heathy human volunteers. Neutrophils release properdin upon activation and are an important source of serum properdin (29). Activated neutrophils release properdin stored within secondary granules (22) into the local, cellular microenvironments, which may contribute to the overall inflammatory response within these environments. However, the distribution of neutrophil-derived properdin oligomers (which would correlate to properdin function) is unknown. To address this gap, the function between serum-derived and neutrophil-derived properdin was assessed. To carry out a comparison at the individual level, serum and neutrophils from the same donor were collected. The neutrophils were activated using PMA, the cellular supernatants were collected, and the concentration of properdin in the supernatants was determined by using the sandwich ELISA as described in the “Materials and Methods” (section 2.5). The concentration of properdin in serum was between 8-19 μg/ml and 649-923 ng/ml in activated neutrophil supernatant. On average, the concentration of properdin released from the cellular supernatant of non-activated neutrophils was 170 +/- 128 ng/ml (data not shown). The function of properdin in serum and in the neutrophil supernatants were next assessed at equivalent properdin concentrations in the functional ELISA as described in the “Materials and Methods” (section 2.8.2). The function for neutrophil-derived properdin was significantly lower (an average 5-fold lower) than serum-derived properdin (**Figure 8**). Given that properdin function quantified in the functional ELISA was a property of oligomerization (**Figure 4**), we next assessed whether the lower function associated with neutrophil-derived properdin was due to an alteration in the ratio of properdin oligomers distinct from the approximate 1:2:1 ratio reported for serum-derived properdin.

#### 3.2.2 The function of neutrophil-derived properdin was due to a properdin oligomer ratio distribution that was different from serum, skewed towards dimers

**Figure 9:**
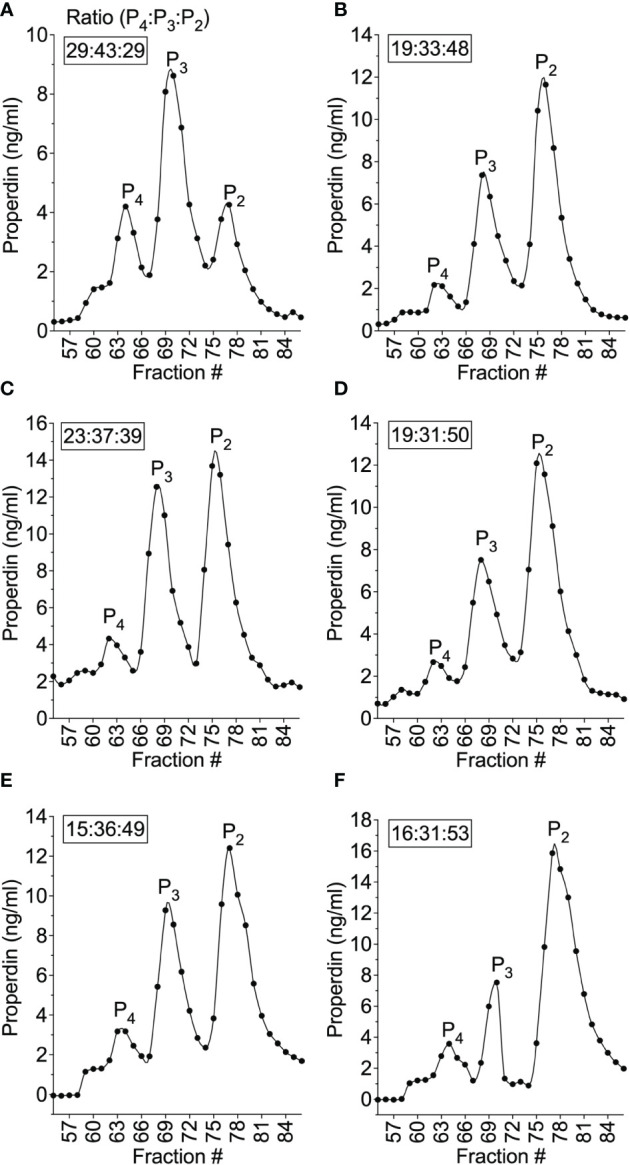
Properdin oligomeric distribution differed between neutrophil-derived and serum-derived properdin and was skewed towards dimers. The ratios of properdin oligomers in serum from donor #4 **(A)** and PMA-activated neutrophil supernatant from donor #4 **(B)**, and PMA-activated neutrophil supernatant from donor #6 **(C)**, #7 **(D)**, #8 **(E)**, #9 **(F)** were determined by size exclusion chromatography and properdin concentration by sandwich ELISA as described in “Materials and Methods” (section 2.5 and 2.6) and the data was graphed by properdin concentration. The ratios of properdin oligomers were calculated as described in “Materials and Methods” (section 2.7). and indicated as an inset to each graph.

To address whether the lower function of neutrophil-derived properdin, as compared to serum, was due to differences in oligomer distribution, size exclusion chromatography coupled with quantification of properdin of the fractions were performed and oligomeric ratios for these samples were calculated as described in the “Materials and Methods” (section 2.7). The ratio of properdin oligomers derived from serum (**Figure 9A**) was an expected ratio of 29:43:29 (P_4_:P_3_:P_2_). The ratio of properdin oligomers in activated neutrophil supernatants from 5 donors (**Figures 9B–F**) was characterized by a lower amount of P_4_/P_3_ and higher amount of P_2_ resulting in oligomeric ratios of 19:33:48, 23:37:39, 19:31:50, 15:36:49, and 16:31:53 (average 18:34:48). Overall, the data indicate that the ratio of properdin oligomers released from PMA-activated neutrophils was different from NHS and favors lower-order forms.

## 4 Discussion

Neutrophils account for a significant source (~19-32%) of circulating properdin (29). It is likely that neutrophils are major contributors of properdin in inflammatory microenvironments, because they are the first cells recruited to sites of infection where they facilitate the clearance of microorganisms *via* phagocytosis, the release of cytotoxic molecules from secondary granules, and the formation of neutrophil extracellular traps (NETs) (reviewed in (30, 31)). Neutrophils also participate in adaptive immune regulation through interactions with antigen-presenting cells and lymphocytes (reviewed in (32)). Neutrophils secrete properdin stored within granules (22) in response to a variety of inflammatory agonists. Properdin carries out a series of essential canonical and non-canonical functions including promotion of: oxidative burst in neutrophils (33), complement activation on NETs (34, 35), complement-mediated platelet-granulocyte formation (20) and also can serve as a ligand for natural killer cells, leading to antimicrobial outcomes independent of complement activation (36). Properdin released from neutrophils can bind activated platelets (37) and apoptotic T-cells (38). Any of these functions may affect inflammatory states both in circulation and especially in cellular microenvironments. Because the magnitude of the function of properdin was directly related to the ratio of properdin oligomers (**Figure 4**), identifying the oligomerization state of properdin when it is released from neutrophils becomes essential, yet remained unknown. In this study, we developed an ELISA-based assay that can measure the function of properdin, dependent on the oligomerization state of properdin in the sample, which allowed us to address this question.

Properdin is known to be essential for stabilizing AP convertases; however, properdin has been shown, *via* surface plasmon resonance, to also serve as a focal point for *de novo* convertase assembly *in vitro* (9). This convertase-initiating property was used to develop the properdin functional assay. The assay relied on a non-inhibitory, monoclonal, anti-human properdin antibody (6E9E6) to capture properdin from various sources including pure properdin, serum, and neutrophil supernatant (**Figure 2**). 6E9E6 was specifically chosen given its non-inhibitory nature and the fact that it cannot distinguish between properdin oligomers when assayed at equivalent ng/ml concentrations (24). Thus, this allows the readout of the assay (C3b deposition) to represent properdin function alone, as a property of oligomer sizes present in the sample.

The function of properdin oligomers derived from either pure properdin or from serum was evaluated to validate the functional assay. For this purpose, properdin from both sources was fractionated into oligomeric forms by size exclusion chromatography, followed by quantification of properdin in each fraction (**Figure 3**). As expected, the ratio of properdin oligomers in both sources followed the approximate 1:2:1 ratio of P_4_, P_3_, and P_2_ and pure properdin contained a small peak consisting of non-physiological aggregates (P_n_) that form after repeat freeze/thaw cycles or prolonged storage whereas NHS contained a minor peak prior to P_4_, which may indicate the presence of pentamers or hexamers, which have been identified in NHS by others (14–16, 27, 39). The addition of the sandwich ELISA for determining concentration of the fractions, following properdin oligomeric separation within serum by size exclusion chromatography, became necessary to define the oligomers within the complex mixture of proteins found within serum. The functional properdin ELISA and size exclusion chromatography are complementary techniques. The ELISA served as an initial high-throughput assay that allows testing of several samples (>28, in triplicate, plus appropriate controls) for detection of differences in overall oligomer-related function between samples, while the size exclusion chromatography was run on individual selected samples to confirm oligomer distribution in relation to the ELISA-based function. In addition, the functional properdin ELISA, given its ability to detect function proportional to size while using very small amounts of properdin (ng), may serve as a practical screening tool for assuring absence of contaminating P_n_ in purified properdin preparations.

When assessing the function of properdin oligomers in the functional ELISA, there was a significant increase in C3b deposition between each properdin oligomer (P_2_, P_3_, and P_4_) collected from both purified properdin and serum (**Figure 4**). In both sources of properdin, P_2_ were less active than P_3_, and P_3_ were less active than P_4_. As expected, the function of serum-derived properdin was comparable to the function of P_3_ from purified properdin and P_2_/P_3_ in the context of serum, as serum-derived properdin contains predominantly P_3_, and does not contain non-physiological P_n_ (15, 27). Properdin function was the highest in non-physiological, aggregated properdin (P_n_), followed by unfractionated pure properdin that contains a mixture of physiological and non-physiological oligomers. Although the P_n_ fraction only represents a small amount of the total properdin within unfractionated properdin, there are several characteristics of properdin that indicate P_n_ are highly potent complement activators: (a) P_n_ elute as a minor peak (**Figure 3A**) with an approximate molecular mass greater than 10^6^ Da (27); (b) structural studies indicate convertases are formed at vertices of properdin oligomers (14); (c) the P_n_ fraction, when added to serum, completely consumes complement (15). Altogether, this indicates that P_n_ act similar to an activating surface, likely due to the high, variable number of available vertices and thus, even small amounts of P_n_ in the context of unfractionated properdin may have exponential effects on the resulting C3b deposition.

The assay was further validated by demonstrating that it can detect concentration-dependent functional differences between unfractionated pure properdin and serum-derived properdin (**Figure 5**). Unfractionated pure properdin, which contains highly aggregated oligomers, had higher function, as expected, than properdin derived from serum across different properdin concentrations (50-500 ng/ml). It was evident that at the lower and higher concentrations, the margin of difference between these two properdin sources narrows. Hence, for evaluating properdin function, the concentration at which properdin is prepared during the capture step must be considered. Altogether, the data indicate the convertase-initiating/stabilizing capacity of properdin oligomers increased with size and highlights the feasibility of applying the functional ELISA to detect functional differences between properdin sources and, in turn, determine whether the ratio of properdin oligomers is skewed towards P_2_ or P_4_.

We and others have shown that binding of purified properdin to necrotic cells and zymosan is dose-dependently inhibited when serum is added to the pure properdin (23, 40). Likewise, when necrotic cells and zymosan are incubated with NHS (heat inactivated or in presence of EDTA, to eliminate complement activity), native properdin in the serum does not bind. The functional assay described herein does not rely on the ability of properdin to bind to a surface. Instead, it utilizes a non-inhibitory monoclonal anti-properdin antibody to capture properdin from any sample, followed by washing, which leaves only properdin that has been captured. Thus, the captured properdin provides a site for *de novo* convertase formation when properdin-depleted sera is added in the following step. It remains possible that hypothetical inhibitory factors could be pulled down with the properdin during the capture step. However, **Figure 4C** shows no difference in the level of C3b deposition between properdin oligomers (P_2_ and P_3_) separated from pure properdin or captured from NHS, suggesting the absence of inhibitory factors in serum that would have contributed to differences in properdin function between these two sources. Properdin function is higher in P_4_ captured from NHS when compared to pure properdin (**Figure 4C**), which while unexpected, rules out the presence of inhibitory factors in serum that would contribute to reduced properdin function. To rule out the possibility that properdin was being captured along with C3(H_2_O) or C3 fragments in biological samples such as serum [as described previously (28)], which could potentially interfere in the outcome, the functional properdin ELISA was carried out with C3-depleted serum as a source of properdin and the results were compared to NHS as a properdin source, at equivalent concentrations (**Figure 7**). The deposition of C3b was not significantly different between properdin sources, suggesting that all the observed function was due to *de novo* convertases forming on the captured properdin and not due to the presence of C3b or C3(H_2_O) captured with properdin, which could have accelerated the formation of convertases and/or increased the signal *per se*. This was further supported by the data indicating a similar level of function between serum-derived properdin and pure P_3_ oligomers (**Figure 4A**). The outcome of the properdin functional ELISA was compared to the function in a properdin functional hemolytic assay that uses AP-sensitive E_R_, properdin-depleted sera, and a properdin source (**Figure 6**). Unlike the functional ELISA (**Figure 4**), the hemolytic assay was not able to consistently detect greater function in higher order oligomers (i.e., P_n_>P_4_>P_3_>P_2_), even when the hemolytic assay parameters were varied, including time allotted for complement activation and percentage of properdin depleted serum (data not shown). P_n_ resulted in little activity, which has been previously shown to be due to complement consumption in the fluid phase that does not occur for physiological properdin oligomers (15). This previous study that detected P_n_-mediated complement consumption using neuraminidase-treated sheep erythrocytes, also detected greater activity in P_4_ versus P_3_ and P_3_ versus P_2_ (15), unlike our hemolysis assay. Differences between the method used may, in part, explain the differences in findings. The functional properdin ELISA, does not result in consumption of P_n_ in the fluid phase, because properdin is captured to the solid phase first, then complement proteins (in the context of properdin-depleted sera) are added in a subsequent step to facilitate convertase assembly on the captured properdin, followed by C3b deposition. This contrasts with hemolytic assays in which properdin sources are mixed simultaneously with properdin-depleted sera (complement protein source) and the erythrocytes, permitting complement activation and consumption on properdin aggregates (i.e., P_n_) in the fluid phase instead of properdin mediating surface-driven complement-mediated hemolysis.

Given that neutrophils serve important roles at sites of inflammation and release properdin when activated, the functional assay was applied to characterize the oligomeric ratio of neutrophil-derived properdin. The functional assay demonstrated that neutrophil-derived properdin was associated with a 5-fold reduction in C3b deposition compared to donor matched serum-derived properdin (**Figure 8**). The cause for this was identified using size exclusion chromatography where the data indicated that neutrophils release properdin predominantly as P_2_, while P_3_ and P_4_ levels are reduced as compared to properdin in NHS (**Figures 9A–E**). This is the first study to demonstrate properdin released from neutrophils exist in a ratio distinct from properdin present in healthy serum. In agreement with the notion that properdin-producing cells may secrete properdin at different oligomeric states, a previous study described T-cell-derived properdin as having approximately 100-fold more activity than serum-derived properdin (41), although the reason for this enhanced activity was never reported. Given that properdin is secreted from numerous sources in addition to neutrophils (reviewed in (21)), these data suggest the ratio of properdin oligomeric forms may differ according to cell type, although whether the release of lower order properdin oligomers is unique to neutrophils remains unknown. In addition, neutrophils release properdin in response to numerous activators including bacterial lipopolysaccharide, tumor necrosis factor-α, interleukin-8, granulocyte macrophage colony stimulating factor, C5a, N-formylmethionine-leucyl-phenylalanine (fMLP), PMA, interferon-α, and influenza A virus (33, 42, 43). It remains to be determined if neutrophils stimulated by activators other than PMA leads to the release of properdin oligomers at distinct ratios.

The finding that neutrophils release properdin oligomers as mainly P_2_ is especially relevant in the context of inflammatory microenvironments where changes in the ratio of properdin oligomers may influence AP activity and may thus represent a previously unknown complement regulatory mechanism. Properdin released from neutrophils as predominantly lower order oligomers may be advantageous to maintain a basal, low level AP activity near the site of neutrophil activation in the microenvironment to avoid premature consumption of properdin. This would be due to higher order properdin oligomers, specifically P_4_, being the first oligomeric form to be utilized by AP convertases and preferentially consumed (15), given it has the most vertices to interact with convertases (14) and thus the greatest avidity for surfaces on which convertases are formed (27). Properdin released mainly as P_2_ may avoid its premature consumption and enable it to eventually assemble to the approximate 1:2:1 ratio in serum and carry out its important functions in circulation, in cellular microenvironments, and at cell-cell interfaces.

In general, protein oligomerization states may be affected by changes in (a) protein concentration; (b) physiological conditions including temperature, pH, ionic strength; or (c) molecular signaling by ligand binding or post-translational modifications (reviewed in (44, 45)). Specifically, properdin oligomerization may be influenced by post-translational modifications such as O-linked glycans present in TSR4 (8). Moreover, denaturation with guanidine converts properdin into an equal mix of P_2_:P_3_:P_4_ (1:1:1) oligomers (15, 39). Properdin oligomerization is also sensitive to changes in pH. Properdin exposed to acidic, denaturing conditions (pH 2.3) redistributed to a mixture containing similar ratios of all three physiological oligomers and returned to an approximate 1:2:1 ratio of oligomers upon renaturing at pH 7.4 (15). Acidic pH is characteristic of inflammatory microenvironments, such as the tumor microenvironment (reviewed in (46)), synovial fluid of traumatic and osteoarthritic joints (47), and bacterial biofilms (48). Interestingly, intercellular pH increases when neutrophils are stimulated with C5a (49) or fMLP (50) (also reviewed in (51)) that could potentially influence properdin oligomerization. In our study, the pH of the neutrophil supernatant was neutral, suggesting that it is possible that other factors may influence oligomer state. However, it also suggests that neutrophil oligomer ratio may actually be mostly steady-state and that the combination of different oligomer ratios of properdin derived from different cell sources results in the final approximate 1:2:1 ratio in serum. In agreement with the possibility of consistent ratios, exchange from one oligomer form to another has been reported as slow (19) and that the oligomers are highly stable (15) *in vitro*.

Overall, future studies are warranted to understand the molecular mechanisms responsible for the oligomerization ratio of neutrophil-derived properdin and whether, or how, it assimilates to the approximate 1:2:1 ratio in serum.

In conclusion, our study utilized a novel ELISA-based functional assay to describe a previously unknown phenomenon whereby neutrophil-derived properdin was released in an oligomeric ratio (predominantly P_2_) that was distinct from serum. This knowledge contributes to understanding molecular mechanisms of AP regulation in inflammatory microenvironments.

## Data availability statement

The original contributions presented in the study are included in the article/supplementary material. Further inquiries can be directed to the corresponding author.

## Ethics statement

The studies involving human participants were reviewed and approved by University of Toledo Institutional Review Board (IRB). The patients/participants provided their written informed consent to participate in this study.

## Author contributions

Experiments were designed by SRM, SSM, NG, VF, and were conducted by SRM and SSM. The results were analyzed and interpreted and written into the manuscript by SRM, SSM, NG, and VF. MP provided key technical design contributions. All authors critically reviewed the manuscript. All authors contributed to the article and approved the submitted version.
